# Unusual complication of seroma after ventral hernia mesh repair: Digestive perforation by tacks. A case report

**DOI:** 10.1016/j.ijscr.2018.10.044

**Published:** 2018-11-01

**Authors:** Mohamed Amine Elghali, Salsabil Nasri, Asma Seghaier, Khaireddine Dhouioui, Fehmi Hamila, Sabri Youssef, Rached Letaief

**Affiliations:** Department of Digestive Surgery, Fahat Hached University Hospital, University of Medicine of Sousse, Tunisia

**Keywords:** Laparoscopy, Mesh, Seroma, Tacker, Digestive perforation

## Abstract

Intraperitoneal meshes are actually widely used in ventral hernia repair. They can reduce operative time, parietal prejudice and postoperative pain.

One of the most well-known complications of intraperitoneal mesh is seroma, but it usually subsides without any therapeutic problems.

These meshes can be fixed by tackers, suture or glue. Few complications related to the fixation technique are known.

We report the case of a patient who underwent a laparoscopic mesh repair for incisional hernia. He developed an infected bulky seroma for which he had to undergo medical treatment and percutanous drainage in order to avoid the mesh removal. The evolution seemed to be favorable until the occurrence of an unusual and unexpected complication: a digestive fistula of the small bowel in the seroma cavity via a tack adhering to the intestines.

The possibility of digestive lesions by a tack is reported by some cases reports. It seems most often to be related to a technical problem. In our case, this adhesion seems to be secondary to the pressure exerted by the seroma. The complicated seroma can be conservatively treated to save the mesh. However the delay before deciding to remove the mesh when using tackers for its fixation may be shortened.

## Highlights

• Complicated seromas are rare and can cause therapeutic problems.
• The right timing for mesh removal is delicate to be detected.
• Erosion of the small bowel by the tacks has been reported. It seems to be due to technical problems.
• In our case the pressure exerted by the bulky seroma favored the fistulization of the small bowel.
• This case suggests that the type of mesh fixation may influence the time of its removal.

## 1 Introduction

Seromas represent a frequent incident after ventral hernia repair by intraperitoneal Mesh [1]. That is why they are more and more described since the advent of laparoscopic cures. However, these seromas are considered as a complication only in case of infection or when interventional management is necessary. Serious complications reported in the literature include parietal cellulitis, seroma infection, and mesh infection [2].

We report the case of a patient operated by laparoscopic approach in our University Hospital for an incisional hernia and who presented a giant seroma complicated firstly by infection, then in a second time an unusual complication occurred: a digestive fistula in the seroma cavity. Our case is exposed and written according to the SCARE guidelines [3]. This case illustrates a complication which has not been reported in the series of complicated seromas, and proves the possibility of digestive perforation by the tacks.

## 2 Case report

It’s the case of a 51-year-old patient who had a BMIof 31, operated 8 years ago for umbilical hernia. He was taken care of in our institution for large median incisional hernia whose collar measured about 7 cm. He underwent a laparoscopic ventral hernia repair using a 20 × 25 cm PTFE intraperitoneal mesh. The mesh was fixed to the peritoneum by a titanium non-absorbable tacks (Protack- Covidien ®), which was the only type of material provided in our public institution. The intervention was performed by an expert surgeon in laparoscopic parietal surgery.

The immediate postoperative course was uneventful. The patient was postoperatively discharged on the second day.

**Fig. 1:**
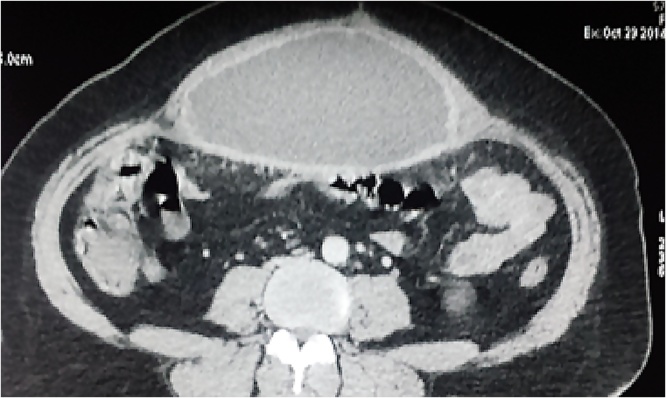
Massive seroma under intraperitoneal mesh.

He was readmitted 8 weeks later for fever and abdominal pain. The physical examination showed a painful and renitent mass next to the surgical site. The biological tests showed leukocytosis at 18,000 el / mm3 and CRP at 120 mg/l. The abdominal Computed Tomography showed a bulky seroma between the mesh and the anterior abdominal wall. (Fig. 1)

Considering the septic syndrome, a percutanous drainage was realized. Two drains were put in place because of the volume of the seroma and to allow later daily washes.

The drains brought back an infected liquid for 3 days then the liquid became clear with a flow rate of 50 ml / day. The bacteriologic exam of the liquid had shown a polymorphic flora. The patient was put on large spectrum antibiotics (third generation of cephalosporin and metronidazole).

The evolution was favorable. The patient was allowed to leave the hospital with the drains in place.

We tried by this attitude to avoid the mesh removal, hoping the spontaneous drying of the drainage.

**Fig. 2:**
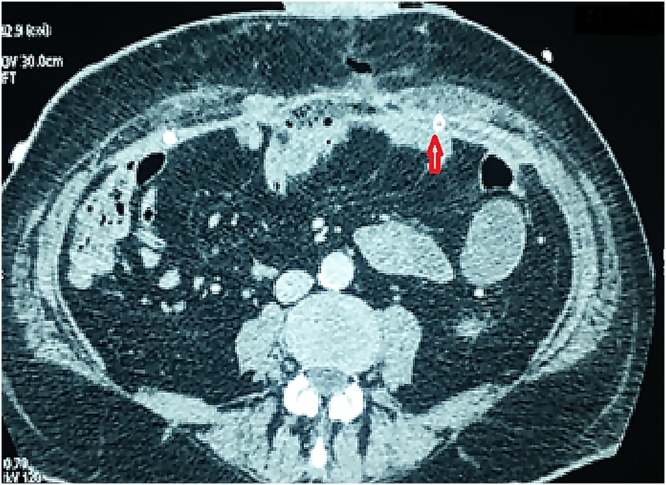
Absence of seroma, the drains are correctly positioned.

A control CT 15 days later did not show a residual collection next to the mesh. The two drains were still in place (Fig. 2). We decided to remove one of them.

**Fig. 3:**
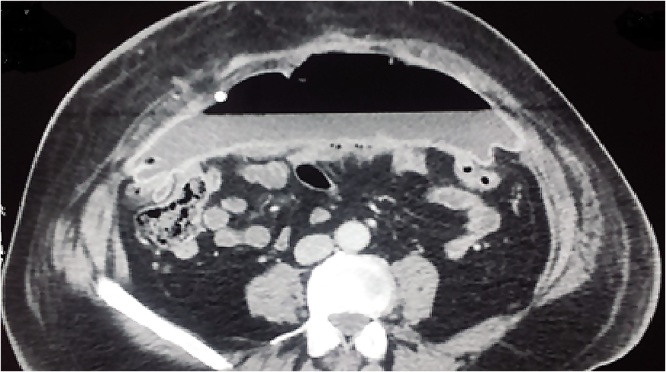
Massive air-fluid level under mesh suggesting digestive fistula.

7 days after the last CT scan (12 weeks post operatively) he was readmitted for severe septic syndrome secondary to an acute peritonitis. The remaining drain brought back a digestive liquid. Abdominal CT found a large air-fluid level between the mesh and the abdominal anterior wall (Fig. 3).

He underwent an emergency laparotomy which showed a bulky purulent collection between the mesh and the anterior abdominal wall. The mesh was easily removed because it was excluded from the peritoneal cavity by a fibrous plane to which it didn’t adhere. After the mesh removal we had discovered an orifice bringing back digestive fluid. The fibrous plane underlying the mesh was dissected allowing the access to the peritoneal cavity. There was no peritonitis below the mesh, but a segment of the small bowel adhered to a tack and was fistulized. Resection-anastomosis was performed. The postoperative course was favorable.

The recurrence of incisional hernia was noted 6 months later, a repair by retro muscular mesh was performed. The evolution was favorable.

## 3 Discussion

Ventral hernia repair is increasingly based on intraperitoneal mesh by laparoscopic approach [4]. This technique causes less parietal damage and less postoperative pain compared to the open approach [5].

Several types of mesh exist, PTFE ones are widely used [6]. The mesh fixation can be done by suture or by tacker. Suture fixation requires a longer operative time.

Seromas after laparoscopic ventral hernia mesh repair are very frequent [1] but they regress spontaneously in almost the majority of cases. Their incidence does not seem to depend on the type of fixation [2]. Complicated seromas requiring interventional management are rare. Their actual incidence is difficult to assess because of the variability of their definition [2]. In our experience their incidence does not exceed 1–2%.

The treatment of bulky seromas, even infected, is intended to save the mesh, so their treatment is based on their drainage and large spectrum antibiotics [2]. In our case, this attitude seemed to give an encouraging result and it is only the occurrence of digestive perforation that precipitated the mesh removal.

The adhesion of the tacks to the small bowel has been reported in the literature [7], but digestive perforation by a tack is extremely rare [8]. This accident may be due to the migration of the tack [9]. In other published cases it is not clear if this accident is secondary or not to a technical defect or if it is a risk inherent to the material.

In our case the pressure exerted by the seroma seems to favor the adhesion of the small bowel to the tack.

This suggests that probably any bulky, even not infected seroma should be routinely drained to minimize the risk of tack adherence to the small bowel. This, in addition, to the systematic precautions of tack on lay: checking its location at the end of the intervention, the absence of digestive wounds and the complete integration of the tacker in the abdominal wall [10].

The therapeutic attitude to preserve the prosthesis in the case of an infected seroma should not be questioned, but the delay before deciding the removal of the prosthesis may be shortened.

## Conflict of interest

Authors declare that there is no conflict of interest.

## Sources of funding

This is not applicable for our manuscript.

## Ethical approval

Exemption from ethnical approval.

## Consent

The patient's consent for the publication was solicited.

## Author contribution

Mohamed Amine Elghali: Writing the paper.

Salsabil Nasri: Writing the paper.

Asma Sghaier: Data analysis.

Khaireddine Dhouioui: Data collection.

Fehmi Hmila: Revision.

Sabri Youssef: Bibliography.

Rached Letaief: Revision.

## Registration of research studies

This is not applicable to our case report.

## Guarantor

Mohamed Amine Elghali.

## Provenance and peer review

Not commissioned, externally peer reviewed.
